# Association of Childhood Infection With IQ and Adult Nonaffective Psychosis in Swedish Men: A Population-Based Longitudinal Cohort and Co-relative Study

**DOI:** 10.1001/jamapsychiatry.2017.4491

**Published:** 2018-02-14

**Authors:** Golam M. Khandaker, Christina Dalman, Nils Kappelmann, Jan Stochl, Henrik Dal, Kyriaki Kosidou, Peter B. Jones, Håkan Karlsson

**Affiliations:** 1 Department of Psychiatry, University of Cambridge, Cambridge, England; 2 Cambridgeshire and Peterborough National Health Service Foundation Trust, Cambridge, England; 3 Centre for Epidemiology and Community Medicine, Stockholm County Council, Stockholm, Sweden; 4 Department of Public Health Sciences, Karolinska Institutet, Stockholm, Sweden; 5 Department of Neuroscience, Karolinska Institutet, Stockholm, Sweden

## Abstract

**Importance:**

Associations between childhood infection, IQ, and adult nonaffective psychosis (NAP) are well established. However, examination of sensitive periods for exposure, effect of familial confounding, and whether IQ provides a link between childhood infection and adult NAP may elucidate pathogenesis of psychosis further.

**Objectives:**

To test the association of childhood infection with IQ and adult NAP, to find whether shared familial confounding explains the infection-NAP and IQ-NAP associations, and to examine whether IQ mediates and/or moderates the childhood infection-NAP association.

**Design, Setting, and Participants:**

Population-based longitudinal cohort study using linkage of Swedish national registers. The risk set included all Swedish men born between 1973 and 1992 and conscripted into the military until the end of 2010 (n = 771 698). We included 647 515 participants in the analysis.

**Measurement of Exposures:**

Hospitalization with any infection from birth to age 13 years.

**Main Outcomes and Measures:**

Hospitalization with an *International Classification of Diseases *diagnosis of NAP until the end of 2011. At conscription around age 18 years, IQ was assessed for all participants.

**Results:**

At the end of follow-up, the mean (SD) age of participants was 30.73 (5.3) years. Exposure to infections, particularly in early childhood, was associated with lower IQ (adjusted mean difference for infection at birth to age 1 year: –1.61; 95% CI, −1.74 to −1.47) and with increased risk of adult NAP (adjusted hazard ratio for infection at birth to age 1 year: 1.19; 95% CI, 1.06 to 1.33). There was a linear association between lower premorbid IQ and adult NAP, which persisted after excluding prodromal cases (adjusted hazard ratio per 1-point increase in IQ: 0.976; 95% CI, 0.974 to 0.978). The infection-NAP and IQ-NAP associations were similar in the general population and in full-sibling pairs discordant for exposure. The association between infection and NAP was both moderated (multiplicative, β = .006; SE = 0.002; *P* = .02 and additive, β = .008; SE = 0.002; *P* = .001) and mediated (β = .028; SE = 0.002; *P* < .001) by IQ. Childhood infection had a greater association with NAP risk in the lower, compared with higher, IQ range.

**Conclusions and Relevance:**

Early childhood is a sensitive period for the effects of infection on IQ and NAP. The associations of adult NAP with early-childhood infection and adolescent IQ are not fully explained by shared familial factors and may be causal. Lower premorbid IQ in individuals with psychosis arises from unique environmental factors, such as early-childhood infection. Early-childhood infections may increase the risk of NAP by affecting neurodevelopment and by exaggerating the association of cognitive vulnerability with psychosis.

## Key Points

**Question:**

What are the associations between childhood infection, IQ, and adult nonaffective psychosis (NAP)?

**Findings:**

In this population-based longitudinal cohort study of Swedish men, early-childhood infection was associated with subsequent lower IQ and increased risk of NAP. The association between infection and NAP was mediated and moderated by IQ, and the associations were similar in the general population and in full-sibling pairs discordant for exposure.

**Meaning:**

Lower premorbid IQ in individuals with psychosis may arise from unique environmental factors, such as early-childhood infection, and early-childhood infection may increase the risk of NAP, partly by interfering with neurodevelopment and partly by exaggerating the effects of cognitive vulnerability to psychosis.

## Teaser

This population-based longitudinal cohort study of Swedish men examines the association of childhood infection with IQ and adult nonaffective psychosis.

## Introduction

There are 3 extensively replicated epidemiologic observations regarding infection, neurodevelopment, and psychotic disorders. First, impaired neurodevelopment increases risk of psychotic disorders as evidenced by longitudinal studies showing premorbid IQ deficit in individuals who subsequently have a psychotic disorder.^1^ Second, exposure to infections during fetal^9^ and childhood^11^ development is associated with risk of adult psychotic disorders. Third, prenatal/childhood infections can alter neurodevelopment as measured by IQ, school grades, or neurologic soft signs.^14^ However, there are also 3 important questions regarding the association of childhood infections with IQ and risk of psychosis. First, is there a sensitive period in development during which exposure to a serious infection is more harmful? Exposure during early, compared with late, childhood may be more harmful because of greater potential for disruptions in a rapidly developing brain. An early insult can interact with subsequent normal brain maturational events to manifest symptoms of psychosis in adulthood.^20^

Second, is the association between childhood infection and adult psychotic disorders likely to be causal, or could this be explained by shared familial confounding? A 2015 study used co-relative control analyses to show that shared familial confounding is an unlikely explanation for the association between lower premorbid IQ and adult psychotic disorders.^2^ The IQ-psychosis association was similar in the general population and in cousin, half-sibling, and full-sibling pairs with differing IQs.^2^ If there were confounding by shared familial risk factors, the association would be expected to become progressively weaker in groups sharing increasing levels of genes and environment compared with the general population; this was not the case. These findings also suggest that unique (nonshared) environmental risk factors may contribute to lower premorbid IQ in individuals with schizophrenia.^2^ Childhood infection may be such an environmental factor not shared within families. To our knowledge, no study has examined the effect of shared familial confounding on the association between childhood infection and adult psychosis. We are not aware of any independent replication of the co-relative analyses of premorbid IQ and adult psychosis.

Third, does impaired neurodevelopment provide a direct link between childhood infection and adult psychotic disorders? Prospective birth cohort studies suggest that childhood infections are associated with increased risk of schizophrenia in adulthood^14^ and with abnormal neurodevelopment in childhood/adolescence as measured by school grade^14^ or neurological soft signs^17^; however, studies using IQ tests in a general population sample are rare. It is unclear whether lower IQ mediates or moderates the association between childhood infection and adult psychosis.

We have addressed these 3 questions using longitudinal data from the Swedish population to elucidate some of the mechanisms through which childhood infection, a common developmental insult, may increase the risk of psychosis in adulthood. We investigated the associations between childhood infections requiring hospital admission from birth to age 13 years, general intelligence as measured by IQ around age 18 years and, as our outcome, subsequent hospitalization in adulthood with a discharge diagnosis of nonaffective psychosis (NAP). We hypothesized (1) childhood infection and lower premorbid IQ would be associated with adult NAP and exposure to infections in early, compared with late, childhood would be more harmful; (2) the IQ-NAP and infection-NAP associations would be similar in the general population and in cousin, half-sibling, and full-sibling pairs discordant for respective exposures; and (3) IQ would mediate and/or moderate the association between childhood infection and adult NAP.

## Methods

### General Population Registers and Study Sample

The National Patient Register (NPR) was used to identify participants hospitalized with infection and NAP. The NPR includes records of virtually all inpatient care in Sweden since 1973 and psychiatric outpatient visits since 2001. Data on IQ of participants at a mean (SD) age of 18.22 (0.41) years were obtained from the military conscription register. The Medical Birth Registry and the Population and Housing Census data were used for birth-related and sociodemographic data, respectively. Military conscription was mandatory in Sweden for all male citizens (with exclusions) born until 1992. The risk set for the analyses included all Swedish men born between 1973 and 1992 who underwent compulsory military conscription between 1991 and 2010 (n = 771 698). We excluded participants who received a diagnosis of psychosis at/before conscription, emigrated from Sweden, had missing data on IQ/date of conscription, and women who had volunteered to serve in the military, resulting in a final analytic sample of 647 515 men (eFigure 1 in the Supplement). To exclude any prodromal effect, we repeated analysis after excluding 390 patients with NAP who were hospitalized within 2 years of IQ assessment. For co-relative control analysis, we identified siblings and cousins for each participant who were born between 1973 and 1992 and conscripted at around age 18 years. This study was approved by the regional ethical committee in Stockholm. No informed consent was needed from participants because all data were anonymous.

### Assessment of Childhood Infection

The NPR was searched from January 1, 1973, to December 31, 2011, to identify participants hospitalized with any infection during childhood from birth to age 13 years.^21^ Serious infection in early childhood before age 5 years may be particularly harmful as measured by future neurologic outcomes, including sensory/motor function, cognition, and psychological well being.^22^ To examine the sensitive period in adequate detail, for main analyses, we divided the sample into 4 groups based on age at infection (0-1, 1-4, 5-9, and 10-13 years). We also carried out sensitivity analyses by grouping infections in 1-year age bands. For all analyses, the exposed group comprised participants with an infection in that particular age group regardless of exposure in any other age; the unexposed group comprised participants with no infection between birth and age 13 years. See eTable 1 in the Supplement for a list of *International Classification of Diseases *(*ICD*) codes for all infections included in analyses.

### Assessment of IQ

The Swedish military service conscription examination involves a full medical assessment, including general intelligence (using IQ scores), measured by 4 subtests representing verbal, logical, spatial, and technical abilities. The global IQ score, derived from a summation of the 4 subtests, was standardized to give a normally distributed score between 1 and 9, which we transformed into units of the Wechsler Adult Intelligence Scale with a mean (SD) score of 100 (15). The IQ scores obtained at conscription have been used in many previous studies, and the assessment process has been previously described.^23^

### Diagnosis of NAP

The NPR was searched from the inception of *ICD-8* in Sweden on January 1, 1969, until December 31, 2011, for cases of NAP. At the end of follow-up, the mean (SD) age of participants was 30.73 (5.30) years, and the oldest participant was aged 38.96 years. The following diagnoses were defined as NAP: inpatient care for *ICD-10* diagnoses of schizophrenia (F20), schizotypal disorder (F21), delusional disorder (F22), psychotic episode (F23), induced delusional disorder (F24), schizoaffective disorder (F25), other psychoses (F28), and psychosis not otherwise specified (F29) and *ICD-9* and *ICD-8* diagnoses of schizophrenia (295), delusional syndrome (297), and nonorganic psychoses (298) excluding reactive psychoses and excluding depressive and manic psychoses (A and B). In Sweden, *ICD-8* codes were used from 1969 to 1986, *ICD-9* codes from 1987 to 1996, and *ICD-10* codes from 1997 to the present.

### Assessment of Covariates

Winter birth (December to May), migration status (either parent born outside Sweden), parental history of NAP (from NPR), household crowding (ie, when the number of livable rooms minus 1 is greater than the number of people living in the house), and parental highest socioeconomic status when the participant was aged 8 to 12 years were included as potential confounders (eTable 2 in the Supplement).

### Statistical Analysis

Cox regression was used to calculate the hazard ratio (HR) and 95% confidence interval for NAP for those exposed to childhood infection compared with those who were unexposed. Linear regression was used to calculate the mean difference in IQ between those exposed to childhood infection compared with unexposed. Hazard ratios for NAP was calculated for each 1-point change in IQ. Linearity of association between IQ and NAP was tested by including a quadratic term (IQ^2^) in the regression model. We calculated HRs separately for schizophrenia and other NAP and separately for central nervous system (CNS) and non-CNS infections.

Interaction between infection and IQ for the outcome of NAP was tested using both multiplicative and additive logistic regression models. For additive interaction, we estimated the Relative Excess Risk Due to Interaction and computed standard errors and *P* values using the delta method.^24^ We examined whether IQ mediated the infection-NAP association by calculating direct and indirect effects. The indirect effect represents risk of NAP that is due to the effect of infection on IQ (ie, mediation).

For co-relative control analyses, we identified all full-sibling, half-sibling, and first-cousin pairs from our sample. Using Cox proportional hazards models, we performed separate analyses on all full-sibling, half-sibling, and first-cousin pairs that were discordant for IQ score (ie, 2 members of each relative pair were not in the same 9-point band), with a separate stratum for each relative pair. The stratified Cox proportional hazards models provide an HR for IQ that is adjusted for the familial cluster and therefore accounts for shared genetic and environmental risk factors.^2^ The same approach was used for infection and NAP using relative pairs discordant for infection in each age band. We carried out sensitivity analyses by defining discordance as lack of exposure to infection at any age. We repeated co-relative analyses stratifying the sample by age at infection (birth to age 4 years, 5 to 13 years).

## Results

### Baseline Characteristics

Of 647 515 participants, 153 460 (23.70%) were hospitalized with an infection between birth and age 13 years. Those exposed to infection were more likely to live in a crowded household, be born to migrant parents, and have a parental history of NAP (eTable 2 in the Supplement). In total, 4045 participants (0.62%) were hospitalized with a diagnosis of NAP during follow-up, including 1455 participants (0.22%) with schizophrenia and 2590 participants (0.40%) with other NAP.

### Association of Childhood Infection With IQ and NAP

Infections at birth to age 13 years were associated with lower IQ measured around age 18 years and increased risk of NAP subsequently in adulthood. Infection during early, compared with late, childhood was more harmful. Infections at birth to age 1 year or 2 to 4 years, but not 5 to 9 or 10 to 13 years, were associated with increased risk of NAP (Table 1). Infections at birth to age 1, 2 to 4, or 5 to 9 years, but not 10 to 13 years, were associated with lower IQ (Table 2). Evidence for these associations remained after adjusting for potential confounders.

**Table 1.** Association Between Childhood Infection and Risk of Nonaffective Psychoses in Adulthood in a Sample of 647 515 Swedish Men

Age at Infection, y	Exposed to Infection, No. (%)	Unadjusted Analysis		Adjusted Analysis^a^	
		Hazard Ratio (95% CI)	*P* Value	Hazard Ratio (95% CI)	*P* Value
All (0-13)	153 460 (23.70)	1.21 (1.13-1.30)	<.001	1.16 (1.08-1.24)	<.001
0-1	49 127 (7.59)	1.29 (1.15-1.44)	<.001	1.19 (1.06-1.33)	<.01
2-4	84 020 (12.98)	1.15 (1.05-1.26)	<.01	1.11 (1.02-1.22)	.02
5-9	34 266 (5.29)	1.01 (0.88-1.15)	.90	1.01 (0.88-1.15)	.90
10-13	14 979 (2.31)	1.01 (0.83-1.23)	.89	1.02 (0.84-1.24)	.84

^a^ Regression models have been adjusted for household crowding, winter birth, parental socioeconomic status, migration status, and parental history of nonaffective psychoses.

**Table 2.** Association Between Childhood Infection and IQ at Age 18 Years in a Sample of 647 515 Swedish Men

Age at Infection, y	Exposed to Infection		Unexposed to Infection		Unadjusted Analysis		Adjusted Analysis^a^	
	Sample, No. (%)	IQ, Mean (SD)	Sample, No.^b^	IQ, Mean (SD)^b^	Mean Difference (95% CI)	*P* Value	Mean Difference (95% CI)	*P* Value
All (0-13)	153 460 (23.7)	99.18 (15.02)	494 055	100.26 (14.98)	−1.08 (−1.16 to −0.99)	<.001	−0.98 (−1.07 to −0.90)	<.001
0-1	49 127 (7.6)	98.20 (15.10)	494 055	100.26 (14.98)	−1.82 (−1.96 to −1.68)	<.001	−1.61 (−1.74 to −1.47)	<.001
2-4	84 020 (13.0)	99.20 (15.02)	494 055	100.26 (14.98)	−0.72 (−0.83 to −0.61)	<.001	−0.70 (−0.81 to −0.60)	<.001
5-9	34 266 (5.3)	99.49 (15.00)	494 055	100.26 (14.98)	−0.36 (−0.52 to −0.20)	<.001	−0.26 (−0.42 to −0.10)	<.01
10-13	14 979 (2.3)	99.69 (14.90)	494 055	100.26 (14.98)	−0.23 (−0.47 to 0.02)	.07	−0.12 (−0.36 to 0.12)	.31

^a^ Regression models have been adjusted for household crowding, winter birth, parental socioeconomic status, migration status, and parental history of nonaffective psychoses.

^b^ The same control group was used for all analyses (ie, participants with no infection between birth and age 13 years).

Childhood infections were associated with schizophrenia and other NAP broadly in a similar fashion, although the association was marginally stronger for other NAP (eTables 3 and 4 in the Supplement). Sensitivity analyses after grouping infections in 1-year age bands showed that NAP was associated with exposure to infections during the first and third year of life. The effect of infection on IQ tended to decrease gradually with increasing age at infection and was mostly statistically nonsignificant after age 5 years (eTables 5 and 6 and eFigures 2 and 3 in the Supplement). Exposure to CNS and non-CNS infections had a similar effect on IQ, but only non-CNS infections were associated with NAP (eTables 7 and 8 in the Supplement).

### Association Between IQ and NAP

**Figure 1.**
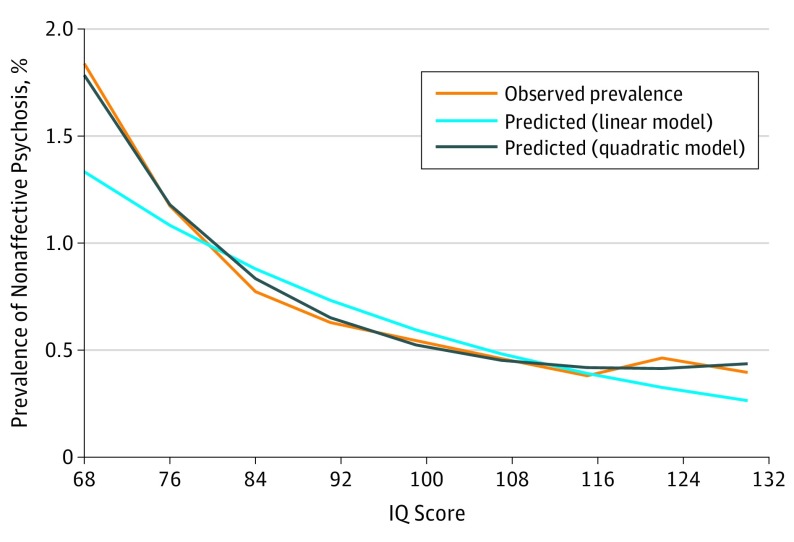
Prevalence of Nonaffective Psychosis as a Function of IQ Score

The risk of NAP declined in a linear fashion across increasing IQ deciles, although the slope of the decline was steeper in the lower compared with the higher IQ range (Figure 1). A linear model fitted to the data produced an HR of 0.975 (95% CI, 0.973-0.977), indicating a 2.5% increase in risk for NAP per 1-point decrease in IQ. We then added a quadratic term to capture the curvilinear function seen in Figure 1, which was statistically significant (χ^2^ = 98.871, *P* < .001).

Exclusion of possible prodromal cases and adjustment for potential confounders had minimal effect on the association between IQ and NAP; adjusted HR per 1-point increase in IQ was 0.976 (95% CI, 0.974-0.978). The effect size for premorbid IQ deficit was similar for schizophrenia and other NAP (eTable 9 in the Supplement).

### Co-relative Control Analysis of IQ and NAP

The association between IQ and NAP was similar in the general population and in cousin, half-sibling, and full-sibling pairs with differing IQs. In the entire sample, the HR for NAP was 0.975 (95% CI, 0.973-0.977). The HRs in co-relative pairs with differing IQs were as follows: HR, 0.972 (95% CI, 0.969-0.975) for first cousin pairs (n = 746 400); HR, 0.981 (95% CI, 0.968-0.995) for half-sibling pairs (n = 46 414); and HR, 0.975 (95% CI, 0.968-0.982) for full-sibling pairs (n = 207 724).

### Co-relative Control Analysis of Childhood Infection and NAP

In the total sample, the infection-NAP association was similar between the general population and full-sibling pairs discordant for infection (Table 3). Likewise, for infection at birth to age 4 years, the HR for NAP was similar between the general population (HR, 1.24; 95% CI, 1.15-1.34) and full-sibling pairs discordant for infection (HR, 1.30; 95% CI, 1.02-1.65). In contrast, there was no evidence for an association between infection at age 5 to 13 years and NAP for any groups (Table 3). The results from sensitivity analysis that defined discordance as lack of exposure to infection at any age were similar (eTable 10 in the Supplement).

**Table 3.** Co-relative Control Analyses of Childhood Infection and Adult Nonaffective Psychoses Based on a Sample of 647 515 Swedish Men

Groups	Birth to Age 13 y at Infection (All)		Birth to Age 4 y at Infection (Sensitive Period)		Age 5 to 13 y at Infection	
	Sample	Hazard Ratio (95% CI)	Sample	Hazard Ratio (95% CI)	Sample	Hazard Ratio (95% CI)
General population	647 515	1.21 (1.13-1.30)	647 515	1.24 (1.15-1.34)	647 515	1.04 (0.92-1.16)
Cousins^a^	304 486	1.08 (0.97-1.20)^b^	250 784	1.04 (0.92-1.17)	119 346	0.99 (0.84-1.17)
Half siblings^a^	21 336	1.03 (0.72-1.48)	18 504	1.00 (0.68-1.46)	8204	1.00 (0.51-1.96)
Full siblings^a^	80 288	1.23 (1.00-1.54)	65 846	1.30 (1.02-1.65)	33 014	0.94 (0.68-1.29)

^a^ Co-relative analyses were based on cousin, half-sibling, and full-sibling pairs who were discordant for infection within each age band (ie, for each relative pair, 1 member was exposed to infection and the other was not exposed to infection during this age band).

^b^ The proportionality assumption for Cox regression was violated for this model, so this particular hazard ratio should be interpreted with care.

### Moderating and Mediating Effects of IQ on the Infection-NAP Association

**Figure 2.**
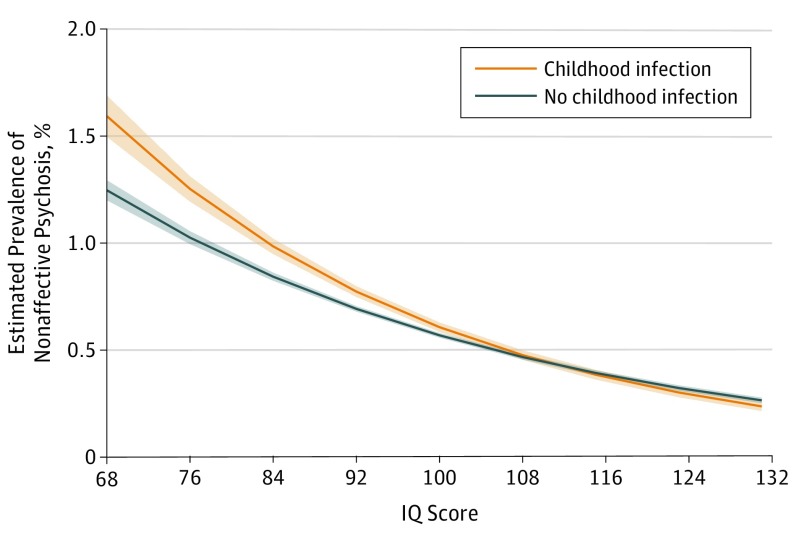
Estimated Prevalence of Nonaffective Psychosis in Groups Exposed and Unexposed to Childhood Infection as a Function of IQ Data is based on multiplicative interaction results from logistic regression moderation analysis.

There was evidence for multiplicative (β = .006; SE = 0.002; *P* = .02) and additive (β = .008; SE = 0.002; *P* = .001) interaction between childhood infection and IQ in the regression models for NAP (eTable 11 in the Supplement). Childhood infection had a greater association with risk of NAP in the lower compared with the higher IQ range (Figure 2). The association between infection and NAP was partly mediated by IQ. The indirect effect (ie, mediated by IQ) of childhood infection on the risk of NAP was statistically significant (β = .028; SE, 0.002; *P* < .001) (eTable 12 in the Supplement).

## Discussion

This study examined potential mechanism of pathogenesis for NAP using childhood infection as exposure and IQ as a marker of neurodevelopment. We report that the effect of infection on IQ and risk of psychosis is most pronounced for exposure during the first year of life (ie, sensitive period). The IQ-NAP association is similar in the general population and in co-relative pairs with discrepant IQs. This indicates that shared familial confounding is unlikely to explain the IQ-NAP association fully. Thus, lower premorbid IQ in individuals with psychosis may arise from unique environmental factors, such as early-childhood infection. To our knowledge, this is 1 of the first studies to use co-relative control analysis to examine familial confounding in the infection-NAP association. Shared familial confounding does not fully explain the association between early-childhood infection and adult NAP, which is consistent with a causal association. Furthermore, IQ mediates and moderates the association between infection and NAP.

The association between IQ and psychosis has been studied extensively.^1^ A meta-analysis of population-based longitudinal studies suggest a linear association between premorbid IQ and schizophrenia.^1^ Findings from our co-relative analysis are consistent with 2 previous Swedish population-based studies.^2^ Our sample has very little overlap with 1 study that included Swedish men born between 1951 and 1975.^2^ Sampling for a second study of Swedish men born between 1971 and 1990 largely overlaps with ours, but it used school achievement as a marker of cognitive performance rather than IQ.^27^

Lower premorbid IQ in patients with schizophrenia may be caused by nongenetic factors related to schizophrenia because genetic variants associated with schizophrenia do not explain variation in IQ in healthy individuals or in patients with schizophrenia.^28^ This is consistent with the findings that lower premorbid IQ is associated with adult NAP independently of genetic relatedness, as reported by Kendler et al^2^ and replicated here. Similarly, polygenic risk scores for schizophrenia are not associated with risk of infections.^29^ Together, these studies suggest that lower premorbid IQ/cognitive performance in cases of NAP may arise from unique environmental factors. We add to this evidence by showing that early-childhood infection could be 1 such factor.

We have examined the interplay between infection and neurodevelopment using moderation and mediation analysis. Childhood infection has a greater association with risk of NAP in the lower, rather than in the higher, IQ range (interaction), suggesting that infections may increase psychosis risk by exaggerating the effects of cognitive vulnerability for psychosis. Statistical evidence for multiplicative and additive interaction is reassuring because statistical interactions are model-dependent.^30^ In a large sample, an analysis may be more likely to find evidence for additive interaction.^30^ Prenatal and childhood infections can interfere with neurodevelopment.^16^ The mediation analysis suggests childhood infections contribute to development of psychosis partly by interfering with neurodevelopment.

We have considered NAP as the main outcome because in early adulthood, it may be more representative of the spectrum of first episode psychotic illness seen in clinical practice, while schizophrenia represents the severe end. We included participants with first hospitalization with a diagnosis of schizophrenia and other NAP. In young people, diagnosis of psychosis can change over time (eg, from other NAP to schizophrenia).^35^ The premorbid IQ deficit was similar for schizophrenia and other NAP indicating that impaired neurodevelopment is relevant for all of these disorders.^2^ Childhood infections were associated with other NAP marginally more strongly, possibly because of a larger number of cases with these diagnoses.

We have analyzed all infections together because population-based, large longitudinal studies suggest that hospitalization with any infection (CNS and other) in childhood is associated with increased risks of schizophrenia and depression in adulthood.^11^ Our sensitivity analysis indicated that non-CNS infections were associated with NAP, which is consistent with previous register-based studies from Denmark^11^ and Sweden.^37^ The lack of an association with CNS infections is not entirely surprising and has previously been reported from these populations.^37^ Although we do not have power to exclude that childhood CNS infections, particularly those occurring in neonatal period, are associated with adult NAP, our current observation adds to an existing literature, suggesting that we should no longer be thinking about the infection-psychosis risk purely in terms of direct damage to the CNS by an infectious agent. Non-CNS infections may contribute to psychiatric risk indirectly (eg, by eliciting systemic inflammation; reviews on how systemic inflammation may influence the brain and risk of schizophrenia have been published^39^). Alternative mechanisms can potentially include CNS involvement from infectious agents typically not considered neurotropic leading to misclassification of exposure^41^ or restrictions in available energy/nutrients owing to competing immune response elicited by a serious infection during periods of rapid growth and development.^42^

### Strengths and Limitations

A large population-based sample, longitudinal design, reliable data, and co-relative analyses are some of the strengths of this study. Limitations include use of admission registers for identifying cases of childhood infection so the findings relate to serious infections only. We have not examined possible effects of treatment. However, as hospitalized cases of infection were included, all participants would have received treatment. There was no association between infection and NAP in discordant half-sibling or cousin pairs who do share some genes and possibly environment. Lack of an association in the half-sibling pairs could be due to a relatively small sample, but this is unlikely to be the case for cousins.

## Conclusions

We present evidence that early childhood is a sensitive period for the effects of infection on IQ and risk of NAP. The associations of adult NAP with early-childhood infection and adolescent IQ are not fully explained by shared familial factors and may be causal. Lower premorbid IQ in NAP arises from unique environmental factors, such as early-childhood infection or other factors intimately related to this. Childhood infection may increase risk of adult NAP by affecting neurodevelopment and by exaggerating the effects of cognitive vulnerability to psychosis.

## Notes

Supplement. **eFigure 1.** Sample Selection for the Analyses of Childhood Infection, IQ, and Nonaffective Psychosis **eFigure 2.** Unadjusted Hazard Ratios for Nonaffective Psychoses for Childhood Infection (sample divided into 1-year age bands based on age at infection) **eFigure 3.** Mean IQ (95% CI) at Conscription for Participants Exposed to Infection in Childhood Grouped by Age at Infection **eTable 1.** ICD Codes for Hospitalized Cases of Childhood Infection **eTable 2.** Baseline Characteristics of Sample **eTable 3.** Association Between Childhood Infection and Risk of Schizophrenia in Adulthood Based on a Sample of 644925 Swedish Men Excluding Cases With Other Nonaffective Psychosis **eTable 4.** Association Between Childhood Infection and Risk of Other Nonaffective Psychosis in Adulthood Based on a Sample of 646060 Swedish Men Excluding Cases with Schizophrenia **eTable 5. **Association Between Childhood Infection and Risk of Nonaffective Psychoses in Adulthood Based on a Sample of 647515 Swedish Men **eTable 6.** Association Between Childhood Infection and IQ at Age 18 Years Based on a Sample of 647515 Swedish Men **eTable 7.** Association Between Childhood CNS and Non-CNS Infections and Risk of Nonaffective Psychoses in Adulthood **eTable 8.** Association Between CNS and Non-CNS Infection in Childhood and IQ at Age 18 **eTable 9.** Hazard Ratio (95% CI) for Schizophrenia and Other Nonaffective Psychosis for Each 1-point Increase in Total IQ Score at Conscription **eTable 10. **Co-relative Control Analyses of Childhood Infection and Adult Nonaffective Psychoses Based on a Sample of 647515 Swedish Men **eTable 11.** Interaction Analyses of Infection and IQ for the Outcome of Nonaffective Psychoses (N=647515) **eTable 12.** Mediating Effect of IQ on the Infection-Nonaffective Psychosis Association (N=647515)
