# Supplementary material for: Association of Childhood Infection With IQ and Adult Nonaffective Psychosis in Swedish Men: A Population-Based Longitudinal Cohort and Co-relative Study
Source: JAMA Psychiatry. 2018 Feb 14;75(4):356–62. doi: 10.1001/jamapsychiatry.2017.4491 (PMC5875340; doi:10.1001/jamapsychiatry.2017.4491)
Supplement: Supplement. — eFigure 1. Sample Selection for the Analyses of Childhood Infection, IQ, and Nonaffective Psychosis eFigure 2. Unadjusted Hazard Ratios for Nonaffective Psychoses for Childhood Infection (sample divided into 1-year age bands based on age at infection) eFigure 3. Mean IQ (95% CI) at Conscription for Participants Exposed to Infection in Childhood Grouped by Age at Infection eTable 1. ICD Codes for Hospitalized Cases of Childhood Infection eTable 2. Baseline Characteristics of Sample eTable 3. Association Between Childhood Infection and Risk of Schizophrenia in Adulthood Based on a Sample of 644925 Swedish Men Excluding Cases With Other Nonaffective Psychosis eTable 4. Association Between Childhood Infection and Risk of Other Nonaffective Psychosis in Adulthood Based on a Sample of 646060 Swedish Men Excluding Cases with Schizophrenia eTable 5. Association Between Childhood Infection and Risk of Nonaffective Psychoses in Adulthood Based on a Sample of 647515 Swedish Men eTable 6. Association Between Childhood Infection and IQ at Age 18 Years Based on a Sample of 647515 Swedish Men eTable 7. Association Between Childhood CNS and Non-CNS Infections and Risk of Nonaffective Psychoses in Adulthood eTable 8. Association Between CNS and Non-CNS Infection in Childhood and IQ at Age 18 eTable 9. Hazard Ratio (95% CI) for Schizophrenia and Other Nonaffective Psychosis for Each 1-point Increase in Total IQ Score at Conscription eTable 10. Co-relative Control Analyses of Childhood Infection and Adult Nonaffective Psychoses Based on a Sample of 647515 Swedish Men eTable 11. Interaction Analyses of Infection and IQ for the Outcome of Nonaffective Psychoses (N=647515) eTable 12. Mediating Effect of IQ on the Infection-Nonaffective Psychosis Association (N=647515) [file jamapsychiatry-75-356-s001.pdf]

Supplementary Online Content

Khandaker GM, Dalman C, Kappelmann N, et al. Association of childhood infection with IQ and adult nonaffective psychosis in Swedish men: a population-based longitudinal cohort and co-relative study. Published online February 14, 2018. *JAMA Psychiatry*. doi:10.1001/jamapsychiatry.2017.4491

eFigure 1. Sample Selection for the Analyses of Childhood Infection, IQ, and Nonaffective Psychosis

eFigure 2. Unadjusted Hazard Ratios for Nonaffective Psychoses for Childhood Infection (sample divided into 1-year age bands based on age at infection)

eFigure 3. Mean IQ (95% CI) at Conscription for Participants Exposed to Infection in Childhood Grouped by Age at Infection

eTable 1. ICD Codes for Hospitalized Cases of Childhood Infection

eTable 2. Baseline Characteristics of Sample

eTable 3. Association Between Childhood Infection and Risk of Schizophrenia in Adulthood Based on a Sample of 644925 Swedish Men Excluding Cases With Other Nonaffective Psychosis

eTable 4. Association Between Childhood Infection and Risk of Other Nonaffective Psychosis in Adulthood Based on a Sample of 646060 Swedish Men Excluding Cases with Schizophrenia

eTable 5. Association Between Childhood Infection and Risk of Nonaffective Psychoses in Adulthood Based on a Sample of 647515 Swedish Men

eTable 6. Association Between Childhood Infection and IQ at Age 18 Years Based on a Sample of 647515 Swedish Men

eTable 7. Association Between Childhood CNS and Non-CNS Infections and Risk of Nonaffective Psychoses in Adulthood

eTable 8. Association Between CNS and Non-CNS Infection in Childhood and IQ at Age 18

eTable 9. Hazard Ratio (95% CI) for Schizophrenia and Other Nonaffective Psychosis for Each 1-point Increase in Total IQ Score at Conscription

eTable 10. Co-relative Control Analyses of Childhood Infection and Adult Nonaffective Psychoses Based on a Sample of 647515 Swedish Men

eTable 11. Interaction Analyses of Infection and IQ for the Outcome of Nonaffective Psychoses (N=647515)

eTable 12. Mediating Effect of IQ on the Infection-Nonaffective Psychosis Association (N=647515)

This supplementary material has been provided by the authors to give readers additional information about their work.

© 2018 Khandaker GM et al. *JAMA Psychiatry.*

eFigure 1. Sample Selection for the Analyses of Childhood Infection, IQ, and Nonaffective Psychosis

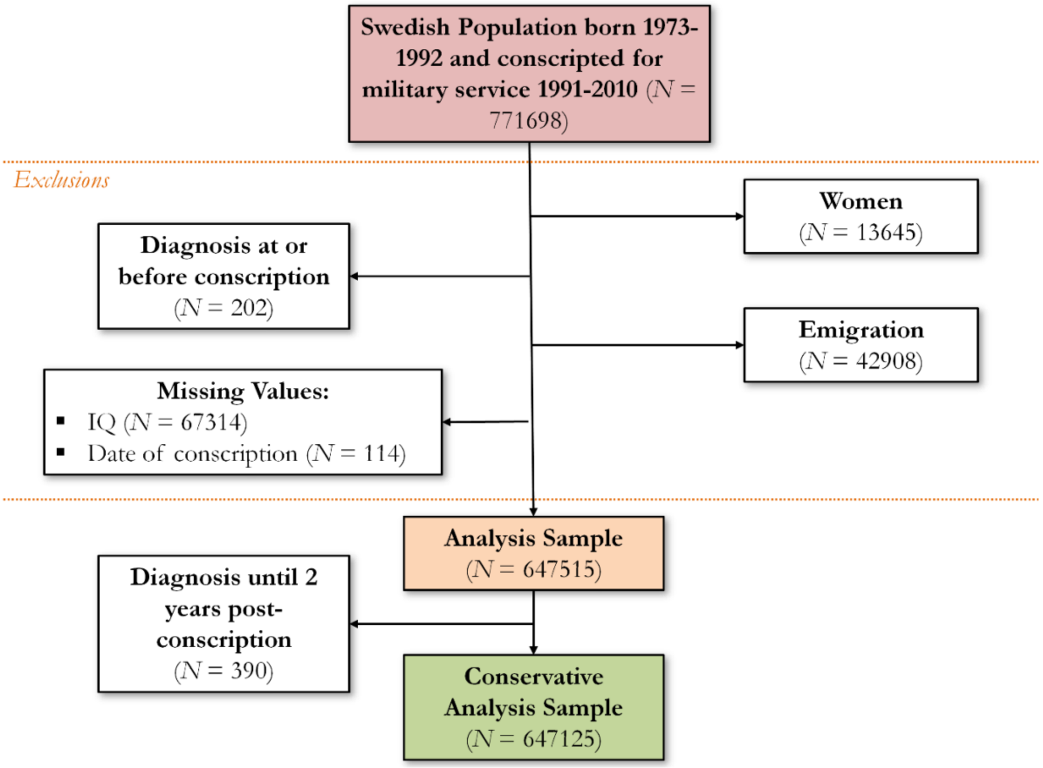

© 2018 Khandaker GM et al. *JAMA Psychiatry.*

eFigure 2. Unadjusted Hazard Ratios for Nonaffective Psychoses for Childhood Infection (sample divided into 1-year age bands based on age at infection)

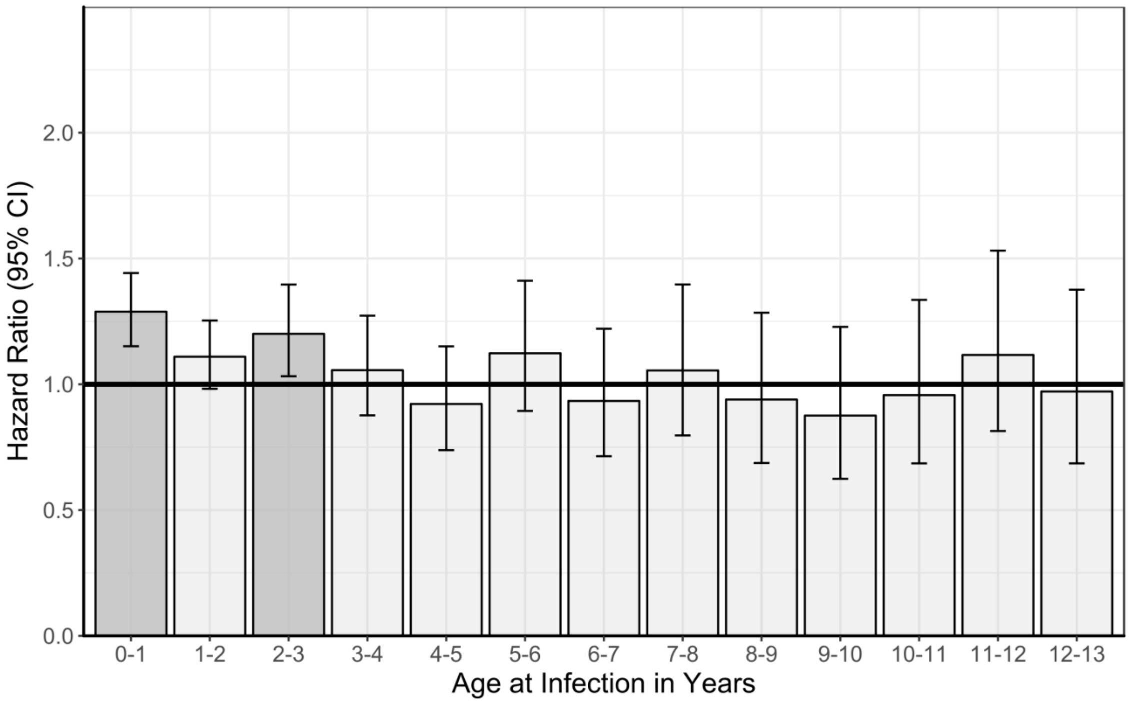

Note: Dark grey bars indicate statistically significant association. The results correspond to unadjusted HR and 95% CI from Cox regression analysis presented in eTable 5.

© 2018 Khandaker GM et al. *JAMA Psychiatry.*

eFigure 3. Mean IQ (95% CI) at Conscription for Participants Exposed to Infection in Childhood Grouped by Age at Infection

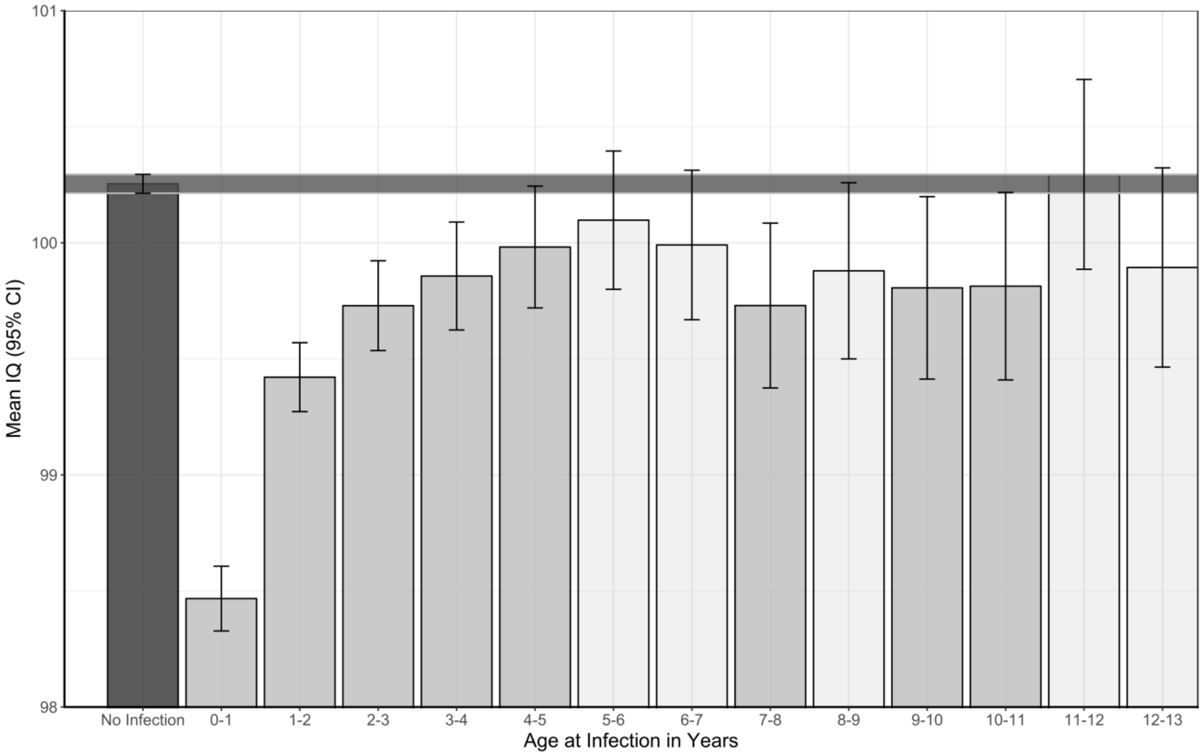

Note: The black bar indicates mean IQ for the unexposed group (i.e., no infection at any age). The grey bars indicate mean IQ for participants exposed to infection grouped by age at infection. Dark grey bars indicate a statistically significant difference in mean IQ for exposure to infection in that particular age compared with unexposed group.

© 2018 Khandaker GM et al. *JAMA Psychiatry.*

eTable 1. ICD Codes for Hospitalised Cases of Childhood Infection

Type of Infection	ICD Version[1]	Diagnostic Codes for Hospitalised Cases of Childhood Infection
Any infection	ICD-8	006.00-007.99, 009.00-009.98, 084.00-087.99, 099.96-099.99, 110.00-130.10, 130.99-131.99, 136.09, 320.88-320.99, 360.00, 380.02-381.99, 384.00-384.08, 420.00-420.09, 421.98, 422.97-422.99, 462.01, 462.09, 463.09, 466.99, 483.99-486.09, 503.00-503.09, 540.00-540.02, 540.04-540.99, 572,99, 686.00-686.98, 761,40, 763.10, 763.98, 778,60 + ICD-8 codes in 'Bacterial infection' and 'Viral infection'.
	ICD-9	006-007X, 008W, 009-D, 084-086X, 099E-X, 110-136X, 321A, 321W, 370E-F, X, 372A-D, 380B, C, 381A, 382X, 420- 422X, 462-463, 466-B, 473-X, 483, 485-486, 490, 491B, 540A, X, 572A, 647C, E, W, X, 680A, 711G-X, 727A, 770A, 771C, E-W + ICD-9 codes in 'Bacterial infection' and 'Viral infection'.
	ICD-10	A06-07.9, A08.5, A09, A59-59.9, A63, A63.8-64, B35-49, B50 -89, B99, G02.1-02.8, G04, G04.9, G05.2, H10.0, H10.3-10.9, H16.2-16.3, H16.9, H32, H60, H60.3, H65.0-65.1, H66.9, I30.0-30.9, I33.0-33.9, I40.0, J02*, J02.8-02.9*, J03*, J03.8-03.9*, J16, J16.8, J18-18.9, J20, J20.8-21, J21.8-21.9, J22, J32-32.9*, J35.0, J37-37.1*, J40-42, K35, K35.9, K75.0, L30.3, M46.5, M65.1, M71.1, O98.3, O98.6-98.9, P23.8-23.9, P37.1-39.9, Z22.4, Z22.8-22.9 + ICD-10 codes in 'Bacterial infection' and 'Viral infection'.
Bacterial	ICD-8	000.01-005.99, 008.00-008.30, 010.99-018.98, 020.00-039.98, 073.99, 076.99, 079.30, 080.99-083.99, 088.99-104.98, 320.00-320.80, 322.00-322.03, 361.00-361.09, 362.02, 366.00, 369.00, 380.00-380.01, 382.00-383.99, 390.97-392.99, 421.00, 461.00-461.09, 462.02, 463.01, 481,99-482.98, 501.99, 508.00-508.02, 510.01-510.09, 511.10, 513.99, 522.50, 527.30, 528.00, 528.30, 540.03, 562.00-562.19, 566.00-566.01, 567.00-567.02, 569.00, 577.01, 590.00-590.99, 595.00-595.02, 597.00, 599.02, 611.00, 611.01, 612.01-614.99, 616.00-616.03, 620.00-620.99, 622.00-622.19, 629.40, 630.00-630.09, 635.00-636.09, 645.90-645.91, 670.00-670.09, 678.02, 680.00-682.99, 684.00-684.09, 710.00-710.09, 720.00-720.29, 732.99, 761,00, 763.00, 998.50, 999.30
	ICD-9	001-005X, 008A-F, 010-041X, 073, 076, 078D, J, 790H, 080-083X, 087-099D, 100-104, 245A, 254B, 320-X, 324-X, 360A, 373B, 375D, 376A, 382A-E, 383A-X, 390-392X, 421A, 461-X, 475, 481-482X, 510-X, 511B, 513-B, 522E, H, 526E, 527D, 528A, D, 540B, 562-B, 566, 567-C, 569F, 575A, 590-X, 597A, 595-D, X, 597W, 599A, 611A, 614-F, W-X, 615A, X, 616-X, 634A, 635A, 636A, 637A, 638A, 639A, 646F, G, 647A, B, D, 658E, 659D, 670, 675-B, W-X, 681-686X, 711A, E, 728A, 729E, 730-D, X, 771D, 996G, 998F, 999D
	ICD-10	A00-05.9, A15-17.9, A20-28.9, A30 -58, A65 -79.9, B95-96.8, E06.0, E32.1, G00-00.9, G01, G04.2, G05.0, G06-06.2*, G07, H00.0, H01.0, H04.3, H05.0, H44.0, H60.0-60.1, H66.0-66.4, H70.0-70.9, I00-02.9, J01-01.9*, J02.0, J03.0, J13-15.9, J16.0, J20.0-20.2, J34.0, J36*, J39.0-39.1, J85.1-85.3, J86-86.9*, K04.6-04.7, K05.2, K11.3, K12.2, K14.0, K35.1, K57-57.9, K61-61.4, K63.0, K65.0*, K81.0, K85, L00 -08.9, M00-00.9, M46.3*, M60.0*, M86-86.9*, N10-12*, N13.6*, N15.1, N15.9, N30-30.3*, N30.8-30.9*, N34-34.1*, N39.0*, , N61, N70-76.8*, N98.0, O07.0, O07.5, O08.0, O23-23.9, O41.1, O75.3, O85-86.8*, O91-91.1, O98.0-98.2, P23.1-23.6, P36, P37.0, T80.2, T81.4, T82.6-82.7, T83.5-83.6, T84.5-84.7, T85.7, T88.0, Z22.0-22.3
Viral	ICD-8	008.80-008.98, 040.00-043.99, 045.00-065.99, 067.00-072.09, 074.00-075.09, 078.00-079.20, 079.40-079.99, 099.92, 460.99, 464.01-480,99, 508.03, 761.20, 761.30
	ICD-9	008H-M, 045-066, 070-072X, 074-075, 077-078H, 078W-079X, 279K, 321B-H, 323A, 323C-D, 460, 464-465X, 480-X, 487-W, 647F, G, 711F, 771A, B, 790W
	ICD-10	A08-08.4, A60-60.9, A63.0, A80-89, A90-99, B00-06.0, B06.8-09, B15-19.9, B20-24, B25-34, B97-97.8, G02.0, G05.1, J00, J04-06.9*, J10-11.8, J12-12.9, J20.3-20.7, J21.0, O35.3, O98.4-98.5, P23.0, P35, Z21, Z22.5-22.6

© 2018 Khandaker GM et al. *JAMA Psychiatry.*

CNS	ICD-8	013.00-013.99, 027.01, 036.00, 090.40, 094.00-094.98, 320.00-320.80, 322.00-322.03, 392.99, 040.00-043.99, 045.00-046.99, 052.00, 054.04, 062.00-065.99, 071.99, 072.01, 075.02, 079.20, 474.99, 084.00, 320.88-320.99
	ICD-9	013-X, 036A, B, 090E, 094-X, 320-X, 324-X, 392-X, 045-049X, 054D, 052B, 053A, 055A, 056A, 071, 072B, C, 321B-H, 323A, 323C, D, 006F, 321A, 321W
	ICD-10	A02.2 (if G01), A17-17.9, A20.3, A22.8, A32.1, A39.0, A39.8 (if G05), A50.4 (if G05.0 or G01), A51.4 (if G01), A52.1 (if G05.0, G01 or F02.8), A54.8 (if G07 or G01), A69.2 (if G01), G00-00.9, G01, G04.2, G05.0, G06-06.2, G07, I02-02.9, A80-89, B00.3-00.4, B01.0-01.1, B02.0-02.1, B05.0-05.1, B06.0, B26.1-26.2, G02.0, G05.1, B58.2, A06.6, B37.5, B38.4, B43.1, B45.1, B46.1, B50.0, B57.4, B60.2, B69.0, B83.2, G02.1-02.8, G04, G04.9, G05.2
Respiratory	ICD-8	010-012, 020.10, 461.00-461.09, 462.02, 463.01, 481,99-482.98, 501.99, 508.00-508.02, 510.01-510.09, 511.10, 513.99, 460.99, 464.01-464.09, 465.99, 470.99-473.99, 480,99, 508.03, 462.01, 462.09, 463.09, 466.99, 483.99-486.09, 502.00-503.09, 519.92, 490.99-491.09
	ICD-9	010-012W, 031A, 033-034B, 052A, 055B, 112E, 122B, 460-466, 475, 481-482X, 510-X, 511B, 513-B, 480-X, 487-W, 462, 463, 466-B, 473-X, 483, 485, 486, 490, 491B
	ICD-10	A15-16, A20.2, A21.2, A22.1, A31.0, A37, A38, A48.1, B00.2, B01.2, B05.2, B27, B37.1, B39-42, B44, B45.0, B46.0, B58.3, B59, J01-01.9, J02.0, J03.0, J13-15.9, J16.0, J20.0-20.2, J34.0, J36, J39.0-39.1, J85.1-85.3, J86-86.9, J00, J04-06.9, J10-11.8, J12-12.9, J20.3-20.7, J21.0, , J02, J02.8-02.9, J03, J03.8-03.9, J16, J16.8, J18-18.9, J20, J20.8-21, J21.8-21.9, J22, J32-32.9, J35.0, J37-37.1, J40-42
Skin	ICD-8	017.01-017.09, 110-111, 050-057, 680.00-680.90, 681.00-682.99, 684.00-684.09, 686.00-686.98
	ICD-9	017A, 031B, 050-057, 074D, 091D, 110-111, 112D, 681-682X, 683, 684, 685-686X, 680A
	ICD-10	A18.4, A20.0, A22.0, A26.0, A31.1, A32, A36.3, B00-09, B35-36, B37.2, B43.0, B43.2, B45.2, B46.3, B55.1, L00, L01-01.1, L02-02.9, L03-03.9, L04-08.9, L70.0, L30.3
Genitourinary	ICD-8	090-099, 016, 054.02, 590.00-590.99, 595.00-595.02, 597.00, 599.02, 601.00, 604.00, 604.01, 607.30, 611.00, 611.01, 612.01-614.99, 616.00-616.03, 620.00-620.99, 622.00-622.19, 629.40
	ICD-9	016, 054B, 112B, C, 090-099, 131A, 590-X, 597A, 595-D, X, 597W, 599A, 601-D, 603B, 604A, 604X, 607B, C, 608A, E, 611A, 614-F, W-X, 615A, X, 616-X
	ICD-10	A18.0-18.1, A50-64, A70-74, B37.3-37.4, N10-12, N13.6, N15.1, N15.9, N30-30.3, N30.8-30.9, N34-34.1, N39.0, N41-41.3, N43.1, N45.0-45.9, N48.1-48.2, N49-49.9, N61, N70-76.8, N98.0
Gastrointestinal	ICD-8	000-009, 014, 039.92, 127.99, 522.50, 527.30, 528.00, 528.30, 562.00-562.19, 566.00-566.01, 567.00-567.02, 569.00, 577.01, 540.00-540.99, 572,99
	ICD-9	001-009, 123, 123, 127, 129, 014, 522E, H, 526E, 527D, 528A, D, 540B, 562-B, 566, 567-C, 569F, 575A, 540A, X, 572A
	ICD-10	001-009, 123, 123, 127, 129, 014, 522E, H, 526E, 527D, 528A, D, 540B, 562-B, 566, 567-C, 569F, 575A, 540A, X, 572A

[1] In Sweden ICD-8 was used from 1969 to 1986, ICD-9 from 1987 to 1996, and ICD-10 from 1997 to date. All diagnoses of post-infection or sequel are excluded.
[*] The additional codes, B95-97.8, denotes the infecting organism. Bacteria= B95-96.8, Virus= B97-97.8.

© 2018 Khandaker GM et al. *JAMA Psychiatry.*

eTable 2. Baseline Characteristics of Sample

Characteristics	Childhood Infection at 0-13 Years			
	**Exposed**	**Unexposed**	**χ^2-Statistic**	***P*-value**
Sample, No. (%)	153460 (23.70)	494055 (76.30)	-	-
Winter Birth, No. (%)	80166 (52.24)	262241 (53.08)	33.15	<0.001
Household Crowding, No. (%)			1026.10	<0.001
Not overcrowded	84833 (55.28)	295520 (59.82)		
Overcrowded	3183 (2.07)	10275 (2.08)		
Unknown	65444 (42.65)	188260 (38.11)		
Parental Socioeconomic Status, No. (%)			1256.00	<0.001
Worker	28139 (18.33)	95065 (19.24)		
White Collar	46909 (30.57)	166122 (33.62)		
Business owner	8720 (5.68)	33005 (6.68)		
Unknown	69692 (45.41)	199863 (40.45)		
Migrant Parents, No. (%)	22951 (14.96)	64915 (13.14)	329.26	<0.001
Parental History of Non-Affective Psychosis, No. (%)	3076 (2.00)	9027 (1.83)	19.97	<0.001

© 2018 Khandaker GM et al. *JAMA Psychiatry.*

eTable 3. Association Between Childhood Infection and Risk of Schizophrenia in Adulthood Based on a Sample of 644925 Swedish Men Excluding Cases With Other Nonaffective Psychosis

Age At Infection	Exposed to Infection, No. (%)	Unadjusted Analysis		Adjusted Analysis[1]	
		Hazard Ratio (95% CI)	*P*-value	Hazard Ratio (95% CI)	*P*-value
All (0-13 years)	152775 (23.69)	1.16 (1.03-1.30)	0.014	1.13 (1.00-1.27)	0.049
0-1 years	48906 (7.58)	1.27 (1.06-1.53)	0.011	1.22 (1.01-1.47)	0.038
2-4 years	83629 (12.97)	1.00 (0.86-1.17)	0.983	0.98 (0.83-1.14)	0.757
5-9 years	34118 (5.29)	1.06 (0.86-1.32)	0.577	1.05 (0.84-1.30)	0.680
10-13 years	14914 (2.31)	1.05 (0.76-1.45)	0.752	1.05 (0.76-1.45)	0.765

[1] Regression models have been adjusted for household crowding, winter birth, parental socioeconomic status, migration status, and parental history of non-affective psychoses.

© 2018 Khandaker GM et al. *JAMA Psychiatry.*

eTable 4. Association Between Childhood Infection and Risk of Other Nonaffective Psychosis in Adulthood Based on a Sample of 646060 Swedish Men Excluding Cases with Schizophrenia

Age At Infection	Exposed to Infection, No. (%)	Unadjusted Analysis		Adjusted Analysis[1]	
		Hazard Ratio (95% CI)	*P*-value	Hazard Ratio (95% CI)	*P*-value
All (0-13 years)	153088 (23.70)	1.24 (1.14-1.35)	<0.001	1.17 (1.08-1.28)	<0.001
0-1 years	49004 (7.58)	1.30 (1.13-1.50)	<0.001	1.17 (1.02-1.35)	0.028
2-4 years	83833 (12.98)	1.24 (1.11-1.38)	<0.001	1.19 (1.07-1.33)	0.002
5-9 years	34179 (5.29)	0.98 (0.83-1.15)	0.769	0.99 (0.83-1.17)	0.874
10-13 years	14941 (2.31)	0.98 (0.77-1.26)	0.878	1.00 (0.78-1.28)	0.986

[1] Regression models have been adjusted for household crowding, winter birth, parental socioeconomic status, migration status, and parental history of non-affective psychoses.

© 2018 Khandaker GM et al. *JAMA Psychiatry.*

eTable 5. Association Between Childhood Infection and Risk of Nonaffective Psychoses in Adulthood Based on a Sample of 647515 Swedish Men

Age At Infection	Exposed to Infection, No. (%)	Unadjusted Analysis		Adjusted Analysis[1]	
		Hazard Ratio (95% CI)	*P*-value	Hazard Ratio (95% CI)	*P*-value
All (0-13 years)	153460 (23.70)	1.21 (1.13-1.30)	<0.001	1.16 (1.08-1.24)	<0.001
0-1 years	49127 (7.59)	1.29 (1.15-1.44)	<0.001	1.17 (1.06-1.33)	0.003
1-2 years	43491 (6.72)	1.12 (0.98-1.25)	0.095	1.06 (0.94-1.20)	0.326
2-3 years	24727 (3.82)	1.20 (1.03-1.40)	0.018	1.18 (1.01-1.37)	0.034
3-4 years	16831 (2.60)	1.06 (0.88-1.27)	0.566	1.05 (0.87-1.26)	0.630
4-5 years	13072 (2.02)	0.92 (0.74-1.15)	0.472	0.92 (0.74-1.15)	0.484
5-6 years	10049 (1.55)	1.12 (0.89-1.41)	0.318	1.13 (0.90-1.42)	0.285
6-7 years	8534 (1.32)	0.93 (0.71-1.22)	0.616	0.93 (0.71-1.22)	0.614
7-8 years	6979 (1.08)	1.05 (0.80-1.40)	0.709	1.06 (0.80-1.40)	0.684
8-9 years	6086 (0.94)	0.94 (0.69-1.28)	0.695	0.93 (0.68-1.28)	0.667
9-10 years	5659 (0.87)	0.88 (0.62-1.23)	0.442	0.87 (0.62-1.23)	0.435
10-11 years	5349 (0.83)	0.96 (0.69-1.34)	0.796	0.95 (0.68-1.33)	0.777
11-12 years	5209 (0.80)	1.12 (0.81-1.53)	0.494	1.14 (0.83-1.56)	0.413
12-13 years	4721 (0.73)	0.97 (0.69-1.38)	0.870	0.99 (0.70-1.40)	0.936

[1] Regression models have been adjusted for household crowding, winter birth, parental socioeconomic status, migration status, and parental history of non-affective psychoses.

© 2018 Khandaker GM et al. *JAMA Psychiatry.*

eTable 6. Association Between Childhood Infection and IQ at Age 18 Years Based on a Sample of 647515 Swedish Men

	Exposed to Infection		Unexposed to Infection		Unadjusted Analysis		Adjusted Analysis[1]	
Age at Infection	Sample	Mean IQ (SD)	Sample	Mean IQ (SD)	Mean Difference (95% CI)	*P*-Value	Mean Difference (95% CI)	*P*-Value
All (0-13 years)	153460	99.18 (15.02)	494055	100.26 (14.98)	-1.08 (-1.16, -0.99)	<0.001	-0.98 (-1.07, -0.90)	<0.001
0-1 years	49127	98.20 (15.10)	494055	100.26 (14.98)	-1.79 (-1.93, -1.65)	<0.001	-1.58 (-1.71, -1.44)	<0.001
1-2 years	43491	98.91 (15.04)	494055	100.26 (14.98)	-0.83 (-0.98, -0.68)	<0.001	-0.78 (-0.93, -0.64)	<0.001
2-3 years	24727	99.14 (15.09)	494055	100.26 (14.98)	-0.53 (-0.72, -0.33)	<0.001	-0.52 (-0.71, -0.33)	<0.001
3-4 years	16831	99.25 (15.11)	494055	100.26 (14.98)	-0.40 (-0.63, -0.16)	<0.001	-0.36 (-0.59, -0.14)	0.002
4-5 years	13072	99.38 (14.96)	494055	100.26 (14.98)	-0.27 (-0.53, -0.01)	0.042	-0.27 (-0.52, -0.01)	0.043
5-6 years	10049	99.53 (15.12)	494055	100.26 (14.98)	-0.16 (-0.45, 0.14)	0.303	-0.12 (-0.41, 0.17)	0.422
6-7 years	8534	99.46 (15.05)	494055	100.26 (14.98)	-0.26 (-0.59, 0.06)	0.109	-0.13 (-0.44, 0.19)	0.434
7-8 years	6979	99.24 (15.11)	494055	100.26 (14.98)	-0.52 (-0.88, -0.17)	0.004	-0.40 (-0.74, -0.05)	0.025
8-9 years	6086	99.43 (14.96)	494055	100.26 (14.98)	-0.37 (-0.75, 0.00)	0.053	-0.26 (-0.63, 0.11)	0.162
9-10 years	5659	99.38 (14.97)	494055	100.26 (14.98)	-0.45 (-0.84, -0.06)	0.025	-0.36 (-0.74, 0.03)	0.068
10-11 years	5349	99.42 (14.82)	494055	100.26 (14.98)	-0.44 (-0.84, -0.04)	0.032	-0.32 (-0.71, 0.08)	0.117
11-12 years	5209	99.92 (15.07)	494055	100.26 (14.98)	0.04 (-0.37, 0.45)	0.845	0.10 (-0.30, 0.50)	0.632
12-13 years	4721	99.55 (14.83)	494055	100.26 (14.98)	-0.36 (-0.79, 0.07)	0.100	-0.24 (-0.66, 0.18)	0.259

[1] Regression models have been adjusted for household crowding, winter birth, parental socioeconomic status, migration status, and parental history of non-affective psychoses.

© 2018 Khandaker GM et al. *JAMA Psychiatry.*

eTable 7. Association Between Childhood CNS and Non-CNS Infections and Risk of Nonaffective Psychoses in Adulthood

Type of Infection	Sample Size[1]	Exposed to Infection, No. (%)	Unadjusted Analysis		Adjusted Analysis[2]	
			Hazard Ratio (95% CI)	*P*-value	Hazard Ratio (95% CI)	*P*-value
All NAP						
All	647515	153460 (23.70)	1.21 (1.13-1.30)	<0.001	1.16 (1.08-1.24)	<0.001
CNS	498718	4663 (0.93)	1.04 (0.74-1.48)	0.811	1.04 (0.74-1.48)	0.817
Non-CNS	644554	150499 (23.35)	1.22 (1.14-1.31)	<0.001	1.16 (1.08-1.25)	<0.001
Schizophrenia						
All	644925	152775 (23.69)	1.16 (1.03-1.30)	0.014	1.13 (1.00-1.27)	0.049
CNS	496792	4642 (0.93)	1.00 (0.55-1.81)	1.00	0.98 (0.54-1.78)	0.949
Non-CNS	641972	149822 (23.34)	1.16 (1.03-1.30)	0.017	1.12 (1.00-1.27)	0.056
Other NAP						
All	646060	153088 (23.70)	1.24 (1.14-1.35)	<0.001	1.17 (1.08-1.28)	<0.001
CNS	497624	4652 (0.93)	1.07 (0.69-1.16)	0.765	1.08 (0.70-1.66)	0.737
Non-CNS	643108	150136 (23.35)	1.25 (1.15-1.37)	<0.001	1.19 (1.09-1.30)	<0.001

[1] The sample for analysis of CNS infection excludes participants with non-CNS infection and *vice versa*; the sample for analysis of schizophrenia excludes participants with other NAP and *vice versa*.

[2] Regression models have been adjusted for household crowding, winter birth, parental socioeconomic status, migration status, and parental history of non-affective psychoses.

© 2018 Khandaker GM et al. *JAMA Psychiatry.*

eTable 8. Association Between CNS and Non-CNS Infection in Childhood and IQ at Age 18

	Exposed to Infection		Unexposed to Infection		Unadjusted Analysis		Adjusted Analysis[1]	
Type of Infection	Sample	Mean IQ (SD)	Sample	Mean IQ (SD)	Mean Difference (95% CI)	*P*-Value	Mean Difference (95% CI)	*P*-Value
All	153460	99.18 (15.02)	494055	100.26 (14.98)	-1.08 (-1.16, -0.99)	<0.001	-0.98 (-1.07, -0.90)	<0.001
CNS	4663	99.35 (14.68)	494055	100.26 (14.98)	-0.91 (-1.34, -0.47)	<0.001	-0.86 (-1.28, -0.44)	<0.001
Non-CNS	150499	99.17 (15.03)	494055	100.26 (14.98)	-1.09 (-1.17, -1.00)	<0.001	-0.99 (-1.07, -0.90)	<0.001

[1] Regression models have been adjusted for household crowding, winter birth, parental socioeconomic status, migration status, and parental history of non-affective psychoses.

© 2018 Khandaker GM et al. *JAMA Psychiatry.*

eTable 9. Hazard Ratio (95% CI) for Schizophrenia and Other Nonaffective Psychosis for Each 1-point Increase in Total IQ Score at Conscription

Outcome	Sample Size	No. of Cases (%)	Unadjusted Analysis		Adjusted Analysis After Excluding Prodromal Cases[1]	
			Hazard Ratio (95% CI)	*P*-value	Hazard Ratio (95% CI)	*P*-value
All NAP	647515	4045 (0.62)	0.975 (0.973-0.977)	<0.001	0.976 (0.974-0.978)	<0.001
Schizophrenia	644925	1455 (0.22)	0.976 (0.974-0.978)	<0.001	0.976 (0.972-0.980)	<0.001
Other NAP	646060	2590 (0.40)	0.975 (0.973-0.978)	<0.001	0.975 (0.973-0.978)	<0.001

[1] Regression models have been adjusted for household crowding, winter birth, parental socioeconomic status, migration status, and parental history of non-affective psychoses and excluded 390 individuals who were diagnosed with NAP within 2 years of conscription.

© 2018 Khandaker GM et al. *JAMA Psychiatry.*

eTable 10. Co-relative Control Analyses of Childhood Infection and Adult Nonaffective Psychoses Based on a Sample of 647515 Swedish Men

Groups	Sample	Hazard Ratio (95% CI)		
		Age at Infection 0-13 Years (All)	Age at Infection 0-4 Years ("Sensitive Period")	Age at Infection 5-13 Years
General Population	647515	1.21 (1.13-1.30)	1.24 (1.15-1.34)	1.04 (0.92-1.16)
Cousins[1]	304486	1.08 (0.97-1.20)	1.07 (0.94-0.21)	1.00 (0.83-1.21)[2]
Half Siblings[1]	21336	1.03 (0.72-1.48)	0.98 (0.67-1.44)	1.25 (0.58-2.67)
Full Siblings[1]	80288	1.23 (1.00-1.54)	1.32 (1.02-1.71)	1.00 (0.68-1.46)

[1] Co-relative analyses were based on cousin, half-sibling and full-sibling pairs who were discordant for infection (i.e., for each relative pair, one member was exposed to infection and the other was not exposed to infection at any age).

[2] The proportionality assumption for Cox regression was violated for this model, so this particular hazard ratio needs to interpreted with care.

© 2018 Khandaker GM et al. *JAMA Psychiatry.*

eTable 11. Interaction Analyses of Infection and IQ for the Outcome of Nonaffective Psychoses (N=647515)

**Predictor**	**Estimates for Non-Affective Psychosis**		
	**B (SE)**	**OR (95% CI)**	***P*-value**
Intercept	-5.163 (0.019)	-	<0.001
Childhood infection	0.065 (0.040)	1.067 (0.987, 1.153)	0.102
IQ†	0.025 (0.001)	1.025 (1.023, 1.028)	<0.001
Multiplicative Interaction‡	0.006 (0.002)	1.006 (1.001, 1.011)	0.020
RERI	0.008 (0.002)	-	0.001

† IQ has been centred (i.e. Mean=0 and SD=15), and reversed (i.e. OR=effect for each one-point decrease) to allow interpretation of the infection main effects as well as the interaction.
‡ The OR for multiplicative interaction represents risk of non-affective psychoses for every 1-point decrease in IQ for those exposed to infection in addition to the main effects of IQ and infection on psychosis risk.

© 2018 Khandaker GM et al. *JAMA Psychiatry.*

eTable 12. Mediating Effect of IQ on the Infection-Nonaffective Psychosis Association (N=647515)

**Effect of Childhood Infection**	**Estimates for Non-Affective Psychosis, β (SE)**	***P*-value**
Direct Effect	0.106 (0.035)	0.002
Indirect Effect via IQ	0.028 (0.002)	<0.001
Total Effect	0.134 (0.035)	<0.001

© 2018 Khandaker GM et al. *JAMA Psychiatry.*
